# Restricted nasal-only breathing during self-selected low intensity training does not affect training intensity distribution

**DOI:** 10.3389/fphys.2023.1134778

**Published:** 2023-04-20

**Authors:** Ludwig Rappelt, Steffen Held, Tim Wiedenmann, Jan-Philip Deutsch, Jonas Hochstrate, Pamela Wicker, Lars Donath

**Affiliations:** ^1^ Department of Intervention Research in Exercise Training, German Sport University Cologne, Cologne, Germany; ^2^ Department of Movement and Training Science, University of Wuppertal, Wuppertal, Germany; ^3^ Department of Sport and Management, IST University of Applied Sciences, Duesseldorf, Germany; ^4^ Department of Sports Science, Bielefeld University, Bielefeld, Germany

**Keywords:** TID, ventilatory LiT, endurance, rating of perceived exertion, heart rate, blood lactate, power

## Abstract

**Introduction:** Low-intensity endurance training is frequently performed at gradually higher training intensities than intended, resulting in a shift towards threshold training. By restricting oral breathing and only allowing for nasal breathing this shift might be reduced.

**Methods:** Nineteen physically healthy adults (3 females, age: 26.5 ± 5.1 years; height: 1.77 ± 0.08 m; body mass: 77.3 ± 11.4 kg; VO_2_peak: 53.4 ± 6.6 mL·kg^−1^ min^−1^) performed 60 min of self-selected, similar (144.7 ± 56.3 vs. 147.0 ± 54.2 W, *p* = 0.60) low-intensity cycling with breathing restriction (nasal-only breathing) and without restrictions (oro-nasal breathing). During these sessions heart rate, respiratory gas exchange data and power output data were recorded continuously.

**Results:** Total ventilation (*p* < 0.001, η_p_^2^ = 0.45), carbon dioxide release (*p* = 0.02, η_p_^2^ = 0.28), oxygen uptake (*p* = 0.03, η_p_^2^ = 0.23), and breathing frequency (*p* = 0.01, η_p_^2^ = 0.35) were lower during nasal-only breathing. Furthermore, lower capillary blood lactate concentrations were found towards the end of the training session during nasal-only breathing (time x condition-interaction effect: *p* = 0.02, η_p_^2^ = 0.17). Even though discomfort was rated marginally higher during nasal-only breathing (*p* = 0.03, η_p_^2^ = 0.24), ratings of perceived effort did not differ between the two conditions (*p* ≥ 0.06, η_p_^2^ = 0.01). No significant “condition” differences were found for intensity distribution (time spent in training zone quantified by power output and heart rate) (*p* ≥ 0.24, η_p_^2^ ≤ 0.07).

**Conclusion:** Nasal-only breathing seems to be associated with possible physiological changes that may help to maintain physical health in endurance athletes during low intensity endurance training. However, it did not prevent participants from performing low-intensity training at higher intensities than intended. Longitudinal studies are warranted to evaluate longitudinal responses of changes in breathing patterns.

## 1 Introduction

More than 80% of the target training intensities in endurance sports is spent at low aerobic exercise intensities below the first lactate threshold (Seiler, 2010; Stöggl and Sperlich, 2014). For adequate training regulation in elite athletes, different approaches of quantifying training intensity based on oxygen uptake dynamics (Burnley and Jones, 2007) or blood lactate concentrations [e.g., three -zone model (Seiler, 2010)] have been recommended. Following Seiler (2010), training intensity can be categorized into three different zones: A low lactate zone (Low intensity Training, LiT; intensity corresponding to a blood lactate concentration of ≤ 2 mmol·l^−1^), a lactate accommodation zone, where blood lactate production and removal rates maintain an equilibrium (Threshold Training, ThT; intensity corresponding to a blood lactate concentration of 2–4 mmol·l^−1^), and a lactate accumulation zone (High intensity Training, HiT; intensity corresponding to a blood lactate concentration of ≥ 4 mmol·l^−1^), where blood lactate production exceeds maximum clearance rates. The corresponding external [power output (Poole et al., 2016)] or internal [lactate (Beneke et al., 2011), heart rate (Borges et al., 2020), heart rate variability (Rogers et al., 2021) and perceived efforts (Sanders et al., 2017)] load are variables applied to navigate this training stimulus. These differences in exercise intensity determination and application impede conclusive evidence on optimal intensity dependent dose-responsiveness during endurance training scheduling. For example, whether polarized or pyramidal training is more favorable to induce optimal endurance performance improvements is still not elucidated. Although successful endurance athletes complete a particularly large part of their training volume in the LiT zone (Fiskerstrand and Seiler, 2004; Guellich et al., 2009; Seiler, 2010), LiT is frequently performed at slightly higher real training intensities than intended, resulting in a bottom-up-shift towards ThT (Seiler, 2010; Röhrken et al., 2020). In turn, intended HiT is also shifting top-down towards ThT. Consequently, a clear evidence-based differentiation between both training intensity distribution frameworks to justify optimal training stimuli is hampered. Thus, practical strategies should be explored and examined to comply with the intended target exercise training zone.

With regard to training regulation, monitoring the breathing frequency during exercise has been suggested as a potential parameter, mainly as the breathing frequency is strongly associated with the perceived effort during exercise under normal and special conditions (e.g., heat, hypoxia, glycogen-depleted state) (Nicolò et al., 2017b). Furthermore, an increasing work load is associated with an increasing oxygen demand (Gaesser and Poole, 1996). In professional cyclists, the tidal volume (V_T_), breathing frequency (BF) and, subsequently, the total ventilation volume (VE) were found to increase as a function of exercise intensity (Lucía et al., 1999). In this context, it has been reported repeatedly that the nasal contribution to breathing decreases with increasing exercise intensity (Niinimaa et al., 1980; Wheatley et al., 1991; James et al., 1997; Bennet et al., 2003). A turning point from nasal to oronasal breathing has been reported at 38% ± 12% of the predicted maximum physical working capacity for men, and at 55% ± 13% for women in moderately trained young adults (Niinimaa et al., 1980). It is therefore reasonable to assume that athletes breathing exclusively nasally are more likely to comply with lower aerobic exercise intensities (e.g., at an intensity below the first lactate threshold) avoiding the bottom-up trend to ThT.

Against this background, this study examined the effect of nasal-only vs. (non-restricted) oro-nasal breathing during self-selected low intensity cycling on ventilation, power output, oxygen consumption, blood lactate concentration, heart rate response, perceived effort, and perceived discomfort. As restricted nasal-only breathing leads to minimal ventilatory impedance (Tong et al., 2001), this may cause higher perceived efforts. Consequently, athletes may need to adhere to very low exercise intensities to avoid further increases of perceived physical effort. Our findings might help coaches and athletes to guarantee LiT intensities during low intensity training by restricting airway choice.

## 2 Materials and methods

### 2.1 Participants and study design

An *a priori* conducted power analysis [*α* = 0.05, study power (1-*β*-error) = 0.80, r = 0.6, effect size η_p_^2^ = 0.06 (*f* = 0.25)] using g*Power (Version 3.1.9.6) revealed a required a sample size of *n* = 16. Assuming low to moderate (15%–20%) dropouts, *n* = 19 young, and physically healthy adults [3 females, age: 26.5 ± 5.1 years; height: 1.77 ± 0.08 m; body mass: 77.3 ± 11.4 kg; peak oxygen uptake (VO_2_peak): 53.4 ± 6.6 mL·kg^−1^ min^−1^, power at VO_2_peak: 285.7 ± 58.0 W, power (HR) at 2 mmol·l^−1^: 189.5 ± 64.6 W (146.1 ± 14.4 bpm), power (HR) at 4 mmol·l^−1^: 234.7 ± 65.8 W (165.6 ± 10.3 bpm)] were enrolled in this acute randomized controlled crossover trial. Inclusion criteria were i) actively pursuing an endurance sport for at least 2 years (training ≥ 3/week) and ii) no medical condition that potentially impede the completion of all experimental sessions. The study was approved by the local ethical committee (033/2022) and all participants signed an informed written consent prior to start of the study.

The study design for this acute randomized crossover study required three lab visits. The first lab visit consisted of anthropometric evaluations and a step test to determine lactate thresholds and VO_2_peak. During the second and third lab visit, participants performed 60 min of self-selected low-intensity cycling training in a randomized order with either breathing without restriction (oro-nasal) or exclusively nasal breathing (nasal-only). All three lab visits were conducted at least 48 h apart with examinations completed at the same time of day for each participant to avoid circadian interferences. Furthermore, participants were instructed to avoid any strenuous exercise in the 24 h prior to each lab visit. All lab visits were performed individually with a participant to researcher ratio of 1:1.

### 2.2 Testing procedures

To determine individual lactate thresholds and assess VO_2_peak a step test on a concentric cycle ergometer (Wahoo Kickr V5 Fitness WF133, Wahoo Fitness, Atlanta, United States) until voluntary exhaustion was conducted. Cycling was performed with clipless pedals and participants were instructed to permanently remain seated. The test started at a load of 100 W, which was subsequently increased by 20 W every 3 min until exhaustion. Prior to the start of the test, after each 3 min-step and immediately after exercise cessation, blood lactate samples (20 µL) were obtained from the earlobe (Biosen C-Line; EKF Diagnostic Sales, Magdeburg, Germany). Lactate concentrations were subsequently plotted against the load (in W) and fitted with a third order polynomial function. Based on this function, load and heart rate (HR) corresponding to a blood lactate concentration of 2 mmol·l^−1^ and 4 mmol·l^−1^ were determined. Furthermore, throughout the whole test, HR (H9; Polar Electro, Kempele, Finnland) and respiratory gas exchange were continuously recorded breath-by-breath comprising a validated metabolic analyzer (Zan Oxi 600, Zan Messgeräte, Germany). Prior to each measurement, this spirometric system was calibrated, following the manufacturer’s recommendations. V_T_, VE, carbon dioxide output (VCO_2_) and oxygen uptake (VO_2_) were averaged over 30 s. Furthermore, the respiratory exchange ratio (RER) was calculated by dividing VCO_2_ by VO_2_. The highest consecutive oxygen uptake values averaged over 30 s were considered as VO_2_peak. All athletes were verbally encouraged in a standardized manner until exhaustion.

### 2.3 Acute intervention protocol

After the first lab visit, participants received a one-page flyer representing the association between blood lactate rise in dependence of exercise intensity: Based on (Seiler, 2010) three training intensity zones representing low intensity training (LiT; ≤2 mmol·l^−1^), threshold training (ThT, 2–4 mmol·l^−1^), and HiT (> 4 mmol·l^−1^) were indicated by vertical lines. Additionally, a short and easy to understand description of this three-zone-model and the respective training intensities for each training zone was provided beneath the schematic depiction (see Supplementary Material S1 for the original flyer in German and Supplementary Material S2 for an English translation). To ensure that the participants had a sufficient understanding of the term LiT, they were instructed to read this flyer before the second lab visit.

Both training sessions at lab visit 2 and 3 consisted of 60 min of cycling. For the oro-nasal condition, participants were only given the instruction to maintain an intensity corresponding to the LiT training zone as described in the aforementioned flyer and to maintain a steady cadence of ∼80 rpm. Apart from this, they were allowed to choose their training intensity and gearing. Throughout the whole session, HR and respiratory gas exchange were continuously recorded breath-by-breath. Additionally, every 10 min (T10, T20, T30, T40, T50, T60), blood lactate samples were obtained and participants were asked to rate their perceived effort (RPE; CR-10) (Foster et al., 2001) and discomfort (Steele et al., 2016). Furthermore, power data and cycling cadence were recorded at a rate of 1 Hz, which was subsequently downloaded and transferred to a personal computer. Apart from time left in the session, no feedback (i.e., information on HR, lactate concentration, power, or respiratory gas exchange parameters) was provided during the session. For the nasal-only condition, an identical setup was chosen. However, participants were additionally instructed to only breath through their nose. To provide maximal breathing capacity through the nose, 5 min prior to the session multiple sprays of sea water nasal spray were applied per nostril and nasal dilator strips were taped across the bridge of the nose and sides of the nostrils for holding open the anterior nasal aperture. Furthermore, a strip of tape was applied over the mouth to prohibit breathing through the mouth. This tape was accessible and easy to remove for both the researcher and participant in case of an emergency. No significant differences were found in terms of resting VO_2_ [F (1, 18) = 1.79, *p* = 0.20, η_p_^2^ = 0.09 (nasal-only condition: 0.499 ± 0.144 L·min^−1^, oro-nasal condition: 0.469 ± 0.117 L·min^−1^)] and blood lactate concentrations [F (1, 18) = 1.57, *p* = 0.23, η_p_^2^ = 0.08 (restricted: 1.00 ± 0.28 mmol·l^−1^, unrestricted: 1.16 ± 0.48 mmol·l^−1^)] prior to the two training sessions. To determine gross efficiency (GE), the work accomplished was divided by the energy expended and multiplied by 100 to obtain a percentual value (Gaesser and Brooks, 1975). For all further analyses, HR data, respiratory gas exchange data [V_T_, VE, RER, VO_2_, VCO_2_, BF, end tidal pressure of oxygen (PETO_2_), end tidal pressure of carbon dioxide (PETCO_2_)] and ergometer data were averaged over each of the 10 min intervals from T10 to T60. Based on the power output and HR, for each 10-min interval, the percentage of time spent in the respective training zones (LiT, ThT, HiT) was calculated, respectively.

### 2.4 Statistics

Data are presented as mean ± SD. All data were initially assessed for normal distribution and variance homogeneity via visual inspection. For the respective outcome measures (HR, RPE, V_T_, VE, RER, VO_2_, VCO_2_, BF, PETO_2_, PETCO_2_, GE, lactate) separately conducted 2 (condition: oro-nasal vs. nasal-only) × 6 (time: T10, T20, T30, T40, T50, T60) repeated measures of variance (rANOVA) were conducted. To examine “condition” differences (oro-nasal vs. nasal-only) repeated measures of variance (rANOVA) were separately conducted for the respective time spent in training zones (LiT, ThT, HiT). Mauchly’s test for sphericity was performed and, if necessary, Greenhouse-Geisser (GG) corrections were applied. Effect sizes for rANOVA are given as partial eta squared (η_p_^2^) with ≥ 0.01, ≥ 0.06, ≥ 0.14 indicating small, moderate, and large effects, respectively (Cohen, 1988). In case of significant interaction effects, Bonferroni *post hoc* tests were subsequently computed. For pairwise effect size comparison, standard mean differences (SMD) were calculated as differences between means divided by the pooled standard deviations (trivial: | SMD | < 0.2, small: 0.2 ≤ | SMD | < 0.5, moderate: 0.5 ≤ | SMD | < 0.8, large: | SMD | ≥ 0.8) (Cohen, 1988). Statistical analyses were performed using R (version 4.0.5) in its integrated development environment RStudio (version 1.4.1106). A *p*-value below 0.05 was considered as statistically significant.

## 3 Results

### 3.1 Performance-related parameters

**FIGURE 1:**
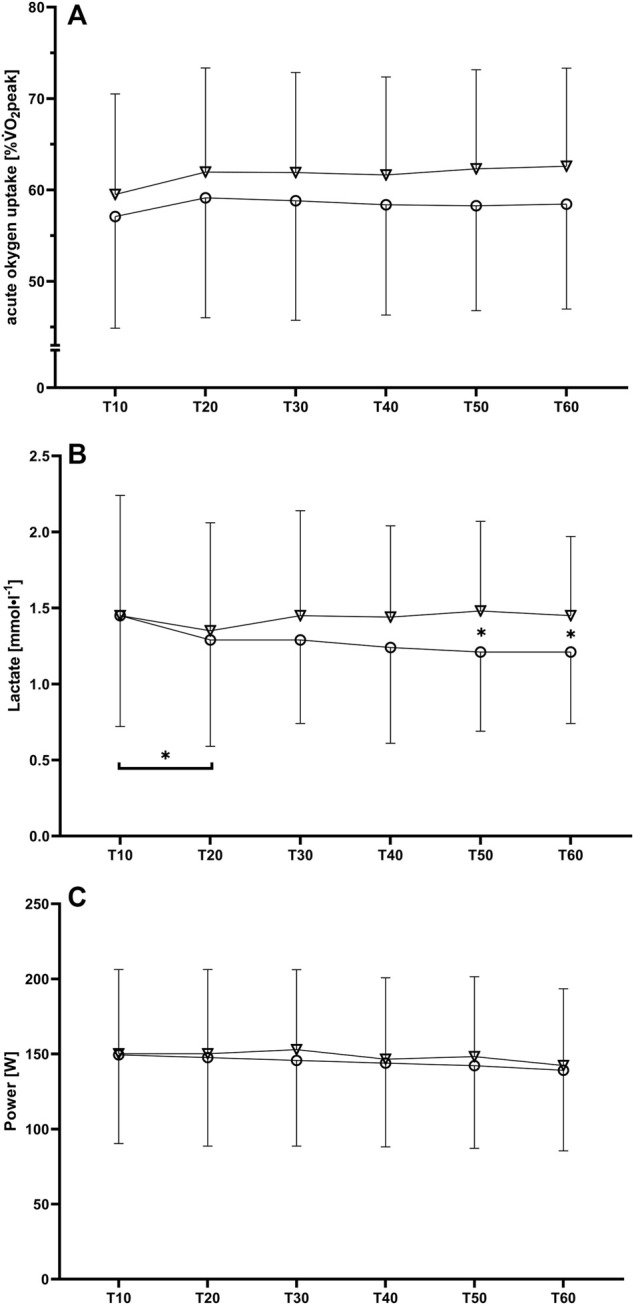
Mean values and standard deviations for **(A)** oxygen uptake related to VO_2_peak, **(B)** blood lactate concentration and **(C)** power output during the six 10-min intervals (T10 to T60) for the nasal-only (circles) and oro-nasal (triangles) training condition. *p < 0.05.

For the acute oxygen uptake related to the participants respective VO_2_peak (%VO_2_peak) no significant interaction effect was found [F (2.5, 44.2) = 1.39, *p* (GG) = 0.26, η_p_^2^ = 0.07], but significant and large main effects for both time [F (1.7, 31.1) = 5.73, *p* (GG) = 0.01, η_p_^2^ = 0.24] and condition [F (1, 18) = 5.49, *p* = 0.03, η_p_^2^ = 0.23] indicating higher values for the oro-nasal condition (Figure 1A). For blood lactate concentrations, a significant and large interaction effect was found [F (3.2, 57.1) = 3.61, *p* (GG) = 0.02, η_p_^2^ = 0.17]. Subsequently performed *post hoc* testing revealed a significant reduction in blood lactate concentration between T10 and T20 in the nasal-only condition (1.45 ± 0.73 vs. 1.29 ± 0.70 mmol·l^−1^, *p* = 0.03, SMD = 0.22). Furthermore, significant differences between the nasal-only and oro-nasal conditions were found for T50 condition (1.21 ± 0.52 vs. 1.48 ± 0.59 mmol·l^−1^, *p* = 0.01, SMD = 0.49) and T60 (1.21 ± 0.47 vs. 1.45 ± 0.52 mmol·l^−1^, *p* = 0.02, SMD = 0.48) (Figure 1B).

No significant interaction effects were found for power [F (1.6, 24.8) = 0.84, *p* (GG) = 0.42, η_p_^2^ = 0.05], cadence [F (2.9, 46.5) = 2.10, *p* (GG) = 0.12, η_p_^2^ = 0.12], and distance [F (1.8, 28.4) = 0.79, *p* (GG) = 0.45, η_p_^2^ = 0.05] (Table 1).

**TABLE 1:** Performance data (mean value ± standard deviation) for the restricted (nasal-only) and unrestricted condition at each 10min interval (T10-T60). *p*-Values and partial eta squared (η_p_^2^) of rANOVA are also provided.

Parameter	Condition	T10	T20	T30	T40	T50	T60	rANOVA *p*-value (η_p_^2^)		
								Time	Condition	Time × condition
Power [W]	Unrestricted	150.1 ± 56.2	150.1 ± 56.1	152.9 ± 53.3	146.6 ± 54.1	148.3 ± 53.2	142.3 ± 51.1	<0.01 (0.37)	0.35 (0.06)	0.42 (0.05)
	Restricted	149.4 ± 59.1	147.6 ± 59.0	145.6 ± 57.0	143.8 ± 55.7	142.2 ± 55.0	139.2 ± 53.7			
Cadence [min^-1^]	Unrestricted	84.7 ± 3.1	87.6 ± 3.1	87.3 ± 3.1	87.4 ± 2.7	88.1 ± 2.8	87.7 ± 4.3	<0.001 (0.44)	0.38 (0.05)	0.12 (0.12)
	Restricted	83.0 ± 2.6	86.7 ± 3.3	87.4 ± 3.5	87.8 ± 3.1	87.9 ± 3.1	86.6 ± 2.9			
Distance [m]	Unrestricted	3,891 ± 902	4,022 ± 947	3,960 ± 924	4,012 ± 937	4,033 ± 920	4,052 ± 908	0.11 (0.14)	0.51 (0.03)	0.45 (0.05)
	Restricted	4,004 ± 972	4,123 ± 965	4,097 ± 969	4,109 ± 963	4,119 ± 992	4,075 ± 987			

Furthermore, the rANOVA did not reveal significant interaction effects for VO_2_ [F (2.3, 42.2) = 1.44, *p* (GG) = 0.25, η_p_^2^ = 0.07], VCO_2_ [F (2.3, 41.5) = 0.67, *p* (GG) = 0.54, η_p_^2^ = 0.04], VE [F (2.3, 41.6) = 1.83, *p* (GG) = 0.17, η_p_^2^ = 0.09], RER [F (2.6, 46.4) = 0.73, *p* (GG) = 0.52, η_p_^2^ = 0.04], BF [F (1.9, 35.0) = 0.31, *p* (GG) = 0.73, η_p_^2^ = 0.02], PETO_2_ [F (2.4, 42.4) = 0.24, *p* (GG) = 0.82, η_p_^2^ = 0.01], PETCO_2_ [F (1.9, 34.8) = 0.26, *p* (GG) = 0.77, η_p_^2^ = 0.01], V_T_ [F (1.9, 34.7) = 0.47, *p* (GG) = 0.62, η_p_^2^ = 0.02], GE [F (1.6, 24.4) = 0.34, *p* (GG) = 0.67, η_p_^2^ = 0.02], HR [F (2.0, 35.2) = 0.41, *p* (GG) = 0.67, η_p_^2^ = 0.02], and discomfort [F (2.6, 47.6) = 2.13, *p* (GG) = 0.12, η_p_^2^ = 0.11], but did reveal significant interaction effects for RPE [F (3.4, 61.8) = 3.38, *p* (GG) = 0.02, η_p_^2^ = 0.16] (Table 2).

**TABLE 2:** Performance data (mean value ± standard deviation) for the restricted (nasal-only) and unrestricted condition at each 10min interval (T10-T60) for oxygen uptake (VO_2_), carbon dioxide release (VCO_2_), total ventilation (VE), respiratory exchange value (RER; VCO_2_ divided by VO_2_), breathing frequency (BF), end tidal pressure of oxygen (PETO_2_), end tidal pressure of carbon dioxide (PETCO_2_), tidal volume (VT), gross efficiency (GE; work accomplished divided by energy expenditure and multiplied by 100), heart rate (HR), perceived effort (RPE), and discomfort. *p*-Values and partial eta squared (η_p_^2^) of rANOVA are also provided.

Parameter	Condition	T10	T20	T30	T40	T50	T60	rANOVA *p*-value (η_p_^2^)		
								Time	Condition	Time × condition
VO_2_ [l·min^−1^]	Unrestricted	2.49 ± 0.74	2.59 ± 0.76	2.59 ± 0.75	2.57 ± 0.74	2.60 ± 0.75	2.61 ± 0.74	0.01 (0.23)	0.03 (0.23)	0.25 (0.07)
	Restricted	2.39 ± 0.78	2.47 ± 0.83	2.46 ± 0.84	2.44 ± 0.79	2.43 ± 0.76	2.44 ± 0.77			
VCO_2_ [l·min^−1^]	Unrestricted	2.19 ± 0.68	2.34 ± 0.72	2.33 ± 0.72	2.29 ± 0.70	2.30 ± 0.69	2.30 ± 0.67	<0.001 (0.46)	0.02 (0.28)	0.54 (0.04)
	Restricted	2.09 ± 0.66	2.23 ± 0.73	2.22 ± 0.73	2.18 ± 0.70	2.16 ± 0.67	2.15 ± 0.67			
VE [l·min^−1^]	Unrestricted	53.0 ± 16.1	57.9 ± 17.9	59.0 ± 19.4	59.2 ± 19.3	59.9 ± 18.6	60.0 ± 18.3	<0.001 (0.68)	<0.001 (0.45)	0.17 (0.09)
	Restricted	48.1 ± 14.2	52.2 ± 15.9	53.1 ± 17.0	53.0 ± 16.6	52.8 ± 15.9	53.0 ± 16.1			
RER [au]	Unrestricted	0.88 ± 0.05	0.90 ± 0.04	0.90 ± 0.05	0.89 ± 0.05	0.88 ± 0.05	0.88 ± 0.05	<0.001 (0.54)	0.67 (0.01)	0.52 (0.04)
	Restricted	0.87 ± 0.05	0.90 ± 0.04	0.90 ± 0.05	0.90 ± 0.05	0.89 ± 0.04	0.88 ± 0.04			
BF [min^−1^]	Unrestricted	26.1 ± 4.4	28.5 ± 4.8	29.7 ± 5.2	30.3 ± 5.2	30.8 ± 5.4	30.7 ± 5.3	<0.001 (0.76)	0.01 (0.35)	0.73 (0.02)
	Restricted	23.3 ± 3.6	25.7 ± 4.0	26.7 ± 4.6	27.2 ± 4.6	27.4 ± 5.0	27.3 ± 4.9			
PETO_2_ [mmHg]	Unrestricted	97.72 ± 4.48	99.92 ± 3.49	100.58 ± 3.68	100.83 ± 3.74	100.85 ± 3.19	100.85 ± 3.14	<0.001 (0.62)	0.01 (0.37)	0.82 (0.01)
	Restricted	95.27 ± 4.96	97.27 ± 4.37	98.39 ± 3.51	98.44 ± 3.60	98.27 ± 3.60	98.17 ± 3.59			
PETCO_2_ [mmHg]	Unrestricted	41.50 ± 2.60	41.05 ± 2.59	40.33 ± 2.91	39.70 ± 2.76	39.31 ± 2.49	39.11 ± 2.36	<0.001 (0.68)	<0.001 (0.46)	0.77 (0.01)
	Restricted	43.47 ± 3.12	43.23 ± 3.59	42.40 ± 3.10	41.87 ± 2.91	41.57 ± 2.82	41.38 ± 2.83			
VT [l]	Unrestricted	2.04 ± 0.56	2.04 ± 0.55	1.99 ± 0.52	1.95 ± 0.49	1.95 ± 0.50	1.95 ± 0.49	<0.001 (0.38)	0.50 (0.03)	0.62 (0.02)
	Restricted	2.11 ± 0.67	2.08 ± 0.70	2.02 ± 0.66	1.98 ± 0.62	1.96 ± 0.62	1.97 ± 0.61			
GE [%]	Unrestricted	17.7 ± 2.1	16.5 ± 1.8	16.4 ± 2.0	16.3 ± 2.1	16.0 ± 2.2	15.7 ± 2.2	<0.001 (0.72)	0.08 (0.20)	0.67 (0.02)
	Restricted	18.3 ± 2.5	17.0 ± 2.0	16.9 ± 2.2	16.9 ± 2.2	16.7 ± 2.3	16.3 ± 2.5			
HR [min^−1^]	Unrestricted	124.2 ± 13.4	130.5 ± 16.2	132.9 ± 17.5	134.3 ± 17.5	136.5 ± 18.0	138.7 ± 18.5	<0.001 (0.77)	0.58 (0.02)	0.67 (0.02)
	Restricted	124.6 ± 16.2	131.4 ± 19.3	133.9 ± 20.0	136.3 ± 21.0	137.6 ± 21.0	139.3 ± 20.7			
RPE [au]	Unrestricted	2.2 ± 0.7^a^	2.6 ± 0.9^c^	2.9 ± 1.0	3.2 ± 1.0	3.4 ± 1.0	3.5 ± 1.0	<0.001 (0.63)	0.75 (0.01)	0.02 (0.16)
	Restricted	2.5 ± 0.8^a^	2.8 ± 0.8^b^	3.0 ± 0.8^d^	3.3 ± 0.8	3.2 ± 0.7	3.2 ± 0.8			
Discomfort [au]	Unrestricted	1.7 ± 0.9	2.1 ± 1.0	2.8 ± 1.1	3.4 ± 1.1	3.8 ± 1.1	4.0 ± 1.2	<0.001 (0.66)	0.03 (0.24)	0.12 (0.11)
	Restricted	2.5 ± 1.6	3.5 ± 1.3	3.7 ± 1.4	3.8 ± 1.7	4.5 ± 1.7	4.9 ± 1.9			

^a^ Significantly different from T30, T40, T50, T60 (*p* < 0.001—0.05).

^b^ significantly different from T40 (*p* < 0.05).

^c^ significantly different from T40, T50, T60 (*p* < 0.001—0.01).

^d^ significantly different from T50, T60 (*p* < 0.05—0.01).

### 3.2 Training zone distribution

**FIGURE 2:**
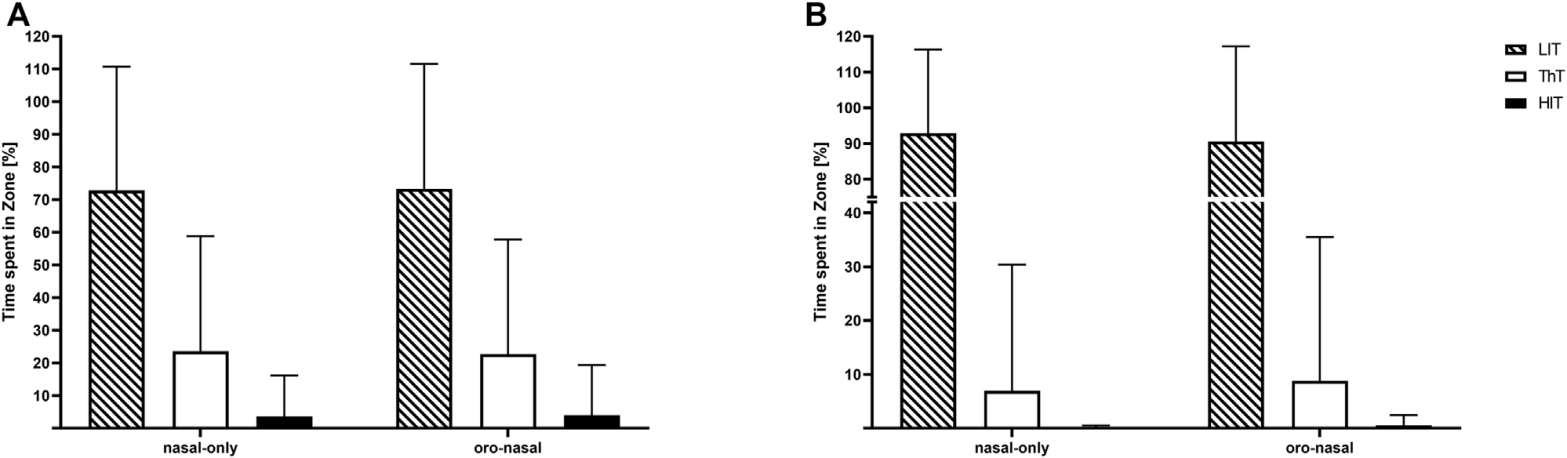
Mean values and standard deviations for the relative time spent in training zones [LiT: low-intensity training (dashed); ThT: threshold training (white); HIT: high-intensity training (black)]. Calculated based on power **(A)** and heart rate **(B)** for the nasal-only and oro-nasal training condition.

The individually conducted 1 x 2 rANOVAs did neither reveal significant “condition” effects for time spent in any of the training zones for power-based calculations of the training zones (Zone 1: (F(1, 18) = 1.45, *p* = 0.24, η_p_^2^ = 0.07); Zone 2: (F(1, 18) = 0.98, *p* = 0.34, η_p_^2^ = 0.05); Zone 3: (F(1, 18) = 1.03, *p* = 0.32, η_p_^2^ = 0.05), Figure 2A) nor heart rate based calculations of the training zones (Zone 1: (F(1, 18) = 0.03, *p* = 0.85, η_p_^2^ = 0.00); Zone 2: (F(1, 18) = 0.14, *p* = 0.71, η_p_^2^ = 0.01); Zone 3: (F(1, 18) = 0.19, *p* = 0.67, η_p_^2^ = 0.01), Figure 2B).

## 4 Discussion

This randomized-controlled crossover trial aimed at investigating the effect of nasal-only vs. oro-nasal breathing during low intensity cycling on power output, oxygen consumption, blood lactate concentration, heart rate, perceived effort and perceived discomfort. No significant differences were found between the two conditions in terms of training intensity outcomes quantified by power output and heart rate. However, total ventilation, carbon dioxide release, oxygen uptake and breathing frequency were notably lower during nasal-only breathing. Furthermore, lower capillary blood lactate concentrations were found towards the end of the training session during nasal-only breathing. These condition-dependent differences between power output and ventilatory response did not affect training intensity distribution (time spent in the three training zones). Interestingly, even though discomfort was rated marginally higher during cycling with nasal-only breathing, ratings of perceived effort did not differ between both conditions.

Our results of lower breathing frequency, total ventilation volume, carbon dioxide release and oxygen uptake during the training session with nasal breathing restriction are in line with previous research on the influence of nasally restricted breathing on cardiorespiratory parameters during continuous submaximal exercise (Morton et al., 1995; Hostetter et al., 2016; LaComb et al., 2017; Recinto et al., 2017). At the same load, similar blood lactate concentrations between nasally restricted and unrestricted breathing conditions have been reported (Dallam et al., 2018). This is also fairly in line with our results, as we did not find increased levels of capillary blood lactate concentration during the nasal-only breathing condition, but even slightly lower values towards the end of the session, which, however, might be related to the decreased power output. It therefore seems plausible, that at least during submaximal exercise intensities the oxygen uptake is not limited by the nasal breathing restriction and thus does not hamper the aerobic energy production. A lower breathing frequency at a given total ventilation volume inherently indicates a higher tidal volume (Harbour et al., 2022), which in turn leads to a reduction in the ratio of the volume of the conducting air passages (anatomic dead space) to the total ventilation volume (Harbour et al., 2022). In the present study, however, even though we found a significantly reduced breathing frequency during the nasal-only condition, the tidal volume was only marginally higher, thus resulting in a lower total ventilation. Moreover, we found a significantly higher end tidal partial pressure of carbon dioxide with a simultaneously lower end tidal partial pressure of oxygen during the nasal-only condition. This may indicate that the lower breathing frequency during nasally restricted breathing leads to a longer pulmonary diffusion time (Morton et al., 1995; Hostetter et al., 2016; LaComb et al., 2017). Therefore, it has been hypothesized that this improvement in ventilatory efficiency during nasal-only breathing at submaximal training intensities may in consequence lead to an improved breathing economy (Hostetter et al., 2016; Dallam et al., 2018). In terms of gross efficiency, however, we did not find significant differences between the two conditions in the present study. It thus seems plausible to assume, that the reduction in minute ventilation is probably due to a reduction in both the breathing frequency and carbon dioxide release.

Despite the lower breathing frequency, oxygen uptake and blood lactate concentration, we did not find any significant and meaningful differences in training intensity distribution between the two conditions. A strong correlation has been frequently reported between the breathing frequency and the perceived effort at moderate to high intensities (Robertson and Noble, 1997; Nicolò et al., 2016; 2017a; 2018; Cochrane-Snyman et al., 2019). In this context, it has been speculated, that a lower breathing frequency might decrease the perceived effort at a certain intensity as the participants are misled to feeling exercise to be easier (Harbour et al., 2022). However, even though we found a statistically significantly lower breathing frequency during the nasal-only breathing condition, perceived effort and power output did not differ between both conditions. In well-trained competitive cyclists, Nicolo and colleagues (Nicolò et al., 2018) reported either no or only small changes in the breathing frequency for given workload intensities corresponding to RPE values of 11 or lower on the 6–20 scale, with considerable changes in breathing frequency at intensities corresponding to > 11 on the RPE scale obtained during sinusoidal tests performed across moderate to severe intensities. It was therefore concluded that the breathing frequency may be considered as sensitive for higher, but not low training intensities (Nicolò et al., 2018). The perceived effort at the first lactate threshold is rated by athletes at 10.4 ± 1.7 on the 6–20 scale (Scherr et al., 2013). This corresponds to the intensity at which the breathing frequency shows a substantial response, which in turn is associated with an increase in perceived effort. Therefore, it seems plausible to assume that the intensity, at which the breathing frequency and subsequently the perceived effort show a substantial response, is located slightly above the first lactate threshold. Thus, this threshold might be too high to be used as a measure to remain in the low-intensity training zone.

A limitation of the study that needs to be addressed is that only the acute effects of a single training session without familiarization to the breathing restrictions were assessed. In this context, the slight decrease in power towards the end of sessions may indicate that perhaps too high a load was selected at the beginning of the sessions. However, as no significant interaction effect was found, and blood lactate concentration did not build up throughout the session in either condition, this seems negligible. Nevertheless, possible longitudinal adaptation to the airway restriction and its effect on the air hunger of the participants should be focused on in future research. Moreover, it might be possible that restricting airway choice may lead to deviations in metabolic thresholds determined during the unrestricted ramp test. However, as demonstrated by Dallam and colleagues (2018), at the same load, similar blood lactate concentrations between nasally restricted and unrestricted breathing conditions can be expected (Dallam et al., 2018).

In terms of long-term adaptations, the diaphragmatic function might increase with time, as during nasal-only breathing a smaller airway is utilized (Trevisan et al., 2015). These adaptations may also help to reduce the higher ratings of perceived discomfort that occurred during nasal-only breathing. Furthermore, the filtration and humidification functions of the nose may help at any exercise intensity to prevent exercise-induced dypnoea and pathogen or particulate inhalation (Aydın et al., 2014). The risk for infections of the upper respiratory tract is significantly reduced when breathing exclusively through the nose during exercise (Walker et al., 2016). By contrast, breathing at submaximal intensities only through the mouth is more likely to cause irritation of the airways, and thus in turn increase the risk of possible exercise-induced laryngeal obstruction (Johansson et al., 2015). Since the head posture and glossopharyngeal mechanics are influenced by different airway choices (Okuro et al., 2011; Sabatucci et al., 2015), breathing predominantly through the nose during submaximal intensities may also prevent exercise-induced laryngeal obstruction (Harbour et al., 2022). Furthermore, by breathing predominantly through the nose, the risk for exercise-induced bronchoconstriction might be reduced (Dallam and Kies, 2020). Therefore, longitudinal studies are necessary to evaluate the long-term effect of nasal-only breathing on perceived effort and physiological parameters in endurance sports.

In conclusion, restricting airway choice did not prevent participants from a tendency to shift from low-intensity training to higher intensities. Nevertheless, temporarily performing low-intensity endurance training under oral breathing restrictions may induce physiological changes that help maintain physical health in endurance athletes.

## Data Availability

The raw data supporting the conclusion of this article will be made available by the authors, without undue reservation.
