# Supplementary material for: Restricted nasal-only breathing during self-selected low intensity training does not affect training intensity distribution
Source: Front Physiol. 2023 Apr 20;14:1134778. doi: 10.3389/fphys.2023.1134778 (PMC10156973; doi:10.3389/fphys.2023.1134778)
Supplement: Supplementary file 1 [file Presentation1.pdf]

Information

3-Zonen-Modell zur Steuerung im Ausdauertraining

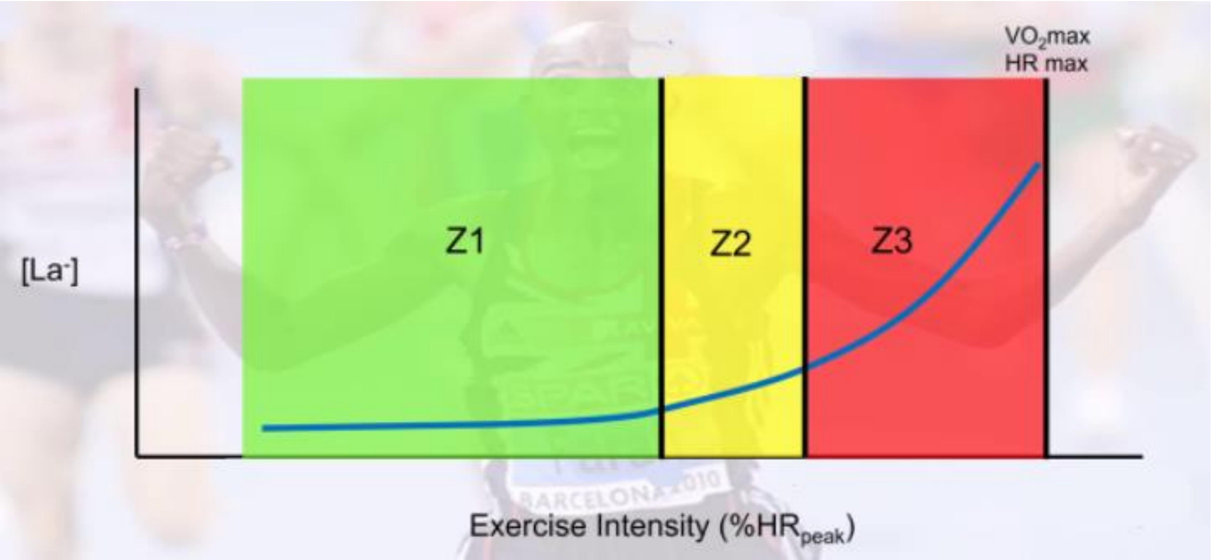

Abbildung 1: Exemplarische Darstellung des 3-Zonen-Modells (Seiler, 2010).

Beim 3-Zonen-Modell wird das Ausdauertraining in drei Intensitätsbereiche eingeteilt. In Abb. 1 ist die Blutlaktatkonzentration auf der y-Achse und die Intensität z.b. Leistung, Geschwindigkeit oder auch prozentualer Anteil der maximalen Herzfrequenz auf der x-Achse abgebildet. Mit steigender Belastung, charakterisiert durch einen Anstieg der Herzfrequenz, steigt die Blutlaktatkonzentration. Anhand des Verlaufs der Laktatkurve lassen sich bestimmte individuelle Trainingszonen bestimmen, welche unter anderem Rückschlüsse auf die Anteile des aeroben und anaeroben Stoffwechsels ermöglichen.

Tabelle 1: Charakterisierung der einzelnen Zonen

**Zone 1 (Z1)**, in der Abbildung in grün hervorgehoben, stellt den niedrigintensiven Bereich dar, welcher durch niedrige Blutlaktatkonzentrationen (ohne nennenswerten Laktat-anstieg) charakterisiert ist. Das Training in Zone 1 wird unter anderem auch als „Low-Intensity Training“ bezeichnet. Belastungen in diesem Trainingsbereich können über mehrere Stunden aufrecht-erhalten werden und sind unterhalb der 1. Laktatschwelle einzuordnen.	**Zone 2 (Z2),** in gelb markiert, beschreibt den Bereich zwischen dem niedrig-intensiven und hochintensiven Bereich. Physiologisch betrachtet bewegen sich Laktatproduktion und -elimination bei diesen Intensitäten im ungefähren Gleichgewicht. Das Training in Zone 2 ist auch unter der Bezeichnung „Schwellen-training“ bekannt und liegt zwischen 1. & 2. Laktat-schwelle.	**Zone 3 (Z3),** der rot markierte Bereich, zeigt den hoch-intensiven Trainings-bereich. Bei Intensitäten in dieser Zone übersteigt die Laktat-konzentration die -elimination. Das Training in Zone 3 wird auch als „High-Intensity Training“ bezeichnet. Diese Intensitäten können über einen relativ kurzen Zeitraum aufrechterhalten werden und liegen oberhalb der 2. Laktat-schwelle.
