# Supplementary material for: Restricted nasal-only breathing during self-selected low intensity training does not affect training intensity distribution
Source: Front Physiol. 2023 Apr 20;14:1134778. doi: 10.3389/fphys.2023.1134778 (PMC10156973; doi:10.3389/fphys.2023.1134778)
Supplement: Supplementary file 2 [file Presentation2.pdf]

Information

3-zone model for endurance training regulation

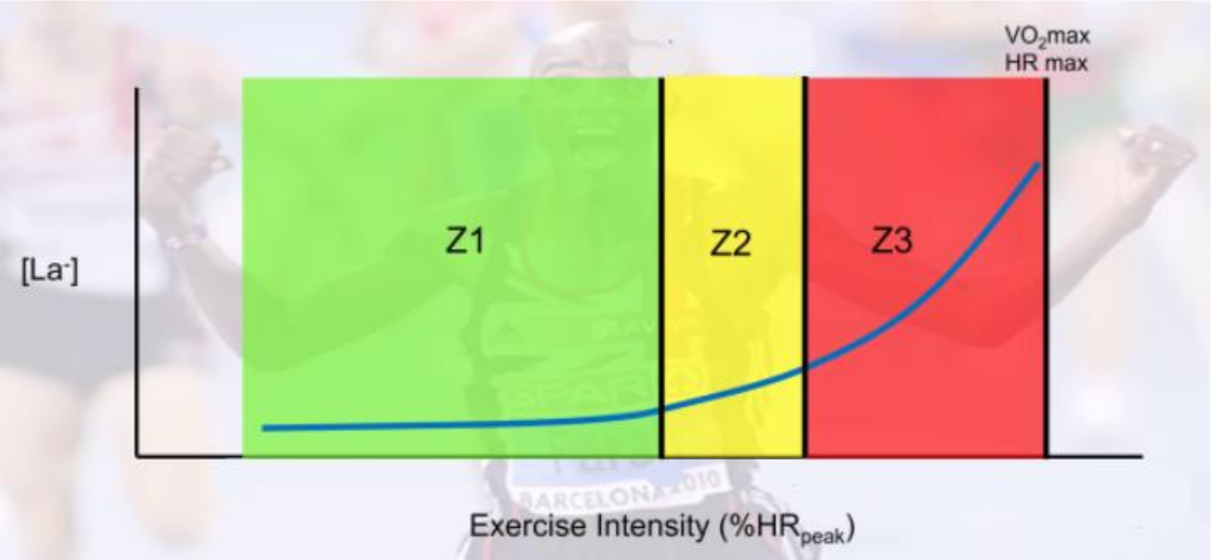

Figure 1: Exemplary representation of the 3-zone model (Seiler, 2010).

According to the 3-zone model, endurance training is divided into three intensity ranges. In Fig. 1, the blood lactate concentration is shown on the y-axis and the intensity, e.g. power, speed or percentage of maximum heart rate, on the x-axis. With increasing load, characterized by an increase in heart rate, the blood lactate concentration rises. Based on the shape of the lactate curve, certain individual training zones can be determined, which, among other things, allow conclusions to be drawn about the substrates being metabolized during exercise.

Table 1: Characteristics of the different training zones.

**Zone 1 (Z1)**, highlighted in green in the figure, represents the low-intensity zone, which is characterized by low blood lactate concentrations (without significant lactate increase). Training in zone 1 is also known as "low-intensity training". Loads in this training zone can be maintained for several hours. Lactate concentration in this zone will remain below the first lactate threshold.	**Zone 2 (Z2),** highlighted in yellow, describes the range between the low-intensity and high-intensity ranges. Physiologically, lactate production and elimination are in an steady-state condition at these intensities. Training in zone 2 is also known as "threshold training" and lies between the first and second lactate threshold.	**Zone 3 (Z3),** highlighted in red, shows the high-intensity training zone. At intensities in this zone, lactate concentration exceeds elimination. Training in zone 3 is also referred to as "high-intensity training". These intensities can be sustained for a relatively short period of time and are above the 2nd lactate threshold.
